# Optical Signatures Derived From Deep UV to NIR Excitation Discriminates Healthy Samples From Low and High Grades Glioma

**DOI:** 10.1038/s41598-019-45181-4

**Published:** 2019-06-19

**Authors:** Hussein Mehidine, Audrey Chalumeau, Fanny Poulon, Frédéric Jamme, Pascale Varlet, Bertrand Devaux, Matthieu Refregiers, Darine Abi Haidar

**Affiliations:** 1 Université de Paris, IMNC Laboratory, UMR 8165-CNRS, IN2P3, Paris, France; 2 Université Paris-Saclay, IMNC Laboratory, UMR 8165-CNRS, IN2P3, Orsay, France; 3 DISCO Beamline, Synchrotron SOLEIL, Gif-sur-Yvette, France; 4 Neuropathology Department, Sainte-Anne Hospital, Paris, France; 5 IMA BRAIN, INSERMU894, Centre de Psychiatrie et de Neurosciences, Paris, France; 6 Paris Descartes University, Paris, France; 7 Neurosurgery Department, Sainte-Anne Hospital, Paris, France

**Keywords:** Cancer imaging, Imaging and sensing

## Abstract

Among all the tumors of the central nervous system (CNS), glioma are the most deadly and the most malignant. Surgical resection is the standard therapeutic method to treat this type of brain cancer. But the diffusive character of these tumors create many problems for surgeons during the operation. In fact, these tumors migrate outside the tumor solid zone and invade the surrounding healthy tissues. These infiltrative tissues have the same visual appearance as healthy tissues, making it very difficult for surgeons to distinguish the healthy ones from the diffused ones. The surgeon, therefore, cannot properly remove the tumor margins increasing the recurrence risk of the tumor. To resolve this problem, our team has developed a multimodal two-photon fibered endomicroscope, compatible with the surgeon trocar, to better delimitate tumor boundaries by relying on the endogenous fluorescence of brain tissues. In this context, and in order to characterize the optical signature of glioma tumors, this study offers multimodal and multi-scaled optical measurements from healthy tissues to high grade glioma. We can interrogate tissue from deep ultra-violet to near infrared excitation by working with spectroscopy, fluorescence lifetime imaging, two-photon fluorescene imaging and Second Harmonic Generation (SHG) imaging. Optically derived ratios such as the Tryptophan/Collagen ratio, the optical redox ratio and the long lifetime intensity fraction, discriminated diseased tissue from its normal counterparts when fitted by Gaussian ellipsoids and choosing a threshold for each. Additionally two-photon fluorescence and SHG images were shown to display similar histological features as Hematoxylin-Eosin stained images.

## Introduction

Brain cancer is one of the deadliest cancers which prevails in humans. Its survival rate is lower than other types of cancer, among ten patients suffering from brain cancer only two survives^1^. Originating from brain neoplastic glial cells, glioma presents 30% of brain tumors and 80% of all malignant brain tumors^2^. They are categorized referring to their location, their cell types and their grades^3^: Low Grade (LG) and High Grade (HG), which is determined by the malignancy and the pathological evaluation of the tumor. This evaluation is performed according to World Health Organization (WHO) classification of CNS tumors^3^. To treat glioma, maximal safe surgical resection is the standard practice used today^4^. The success of this kind of surgery relies on the accurate identification of the solid tumor mass. But actual tumor margins are not well defined as tumor cells diffuse into adjacent normal tissue. This cell diffusion leads to the emergence of infiltrating zones, which overlap with the healthy zones with no distinguishing appearance, thus making their delineation very difficult. The inability to fully visualize the margins of a diffusive glioma results in subtotal surgical resections, which negatively affects the survival rate of the patient^5^ and causes a fast recurrence of the tumor. Similarly, unnecessary removal of healthy functional brain tissue that does not contain cancer cells can lead to major neurological deficits that affect the life quality of the patient.

Most brain cancers are diagnosed by conventional imaging techniques such as computed tomography (CT), magnetic resonance imaging (MRI) and positron emission tomography (PET)^2,6,7^. These imaging techniques characterize the location and the anatomical property of the tumor solid area, but they cannot give structutural or metabolic information at the cellular level. i In addition, these imaging techniques cannot be practically used intraoperatively. Over time, different techniques have been developed to guide the surgery and to improve the surgical outcome like Intra-MRI which gives the surgeon an updated plan on the tumor’s location and its solid boundaries^8^. However, intra-MRI are resource intensive as they require 20–30 minutes for each acquisition, thereby lengthening the duration of the procedure.

A more practical technique that provides real-time information on the tumor boundaries, is the intra-fluorescence guided surgery which uses exogenous fluorescent marquers to acquire fluorescence images^9,10^. Fluorescence guided surgery, using Protoporphyrins IX and its precursor, the 5-Aminolevulinic Acid (5-ALA), increases the extent of tumor resections in patients with Glioblastoma (GBM) (Grade IV glioma) patients^10^, but has shown limited efficacy in identifying the diffuse low-grade gliomas and the micro-infiltration cases^9^. In addition, the fluorescence of this fluorophore is not highly specific to tumors, as PpIX fluorenscence is also present in inflammatory cells within the resection cavity^9^. In addition to this, protoporphyrin IX can escape into peritumoral oedematous areas which are healthy regions that are free of tumor cells. To this end, histological Hematoxylin-Eosin (H&E) staining is still the gold standard for an accurate diagnosis of any brain tissue type. Unfortunately, the results of such methods can only be communicated a few days after the surgery. There is therefore an immense need to develop a more efficient for tissue diagnosis that can be obtained during surgery. This method should be able to sample tissue non-invasively and provide morphological structural and functional information at the tissular, molecular, and cellular level to be sensitive enough to detect isolated diffuse low and high grade glioma cells. One and two-photon excitation can satisfy this need.

Over time, the reliance on endogenous fluorescence has strongly evolved to explore brain tissues, in order to discriminate tumoral from healthy tissues^11–13^ or to discriminate healthy tissues from^13^. The autofluorescence of the Nicotidamine Adeninde Dinucleotide (NADH) and the Flavins (FAD) with its related metabolic ratios shows the importance of getting reliable information on the tissue’s nature a^11–14^. The redox ratio highlights the accretion of NADH in tumor tissues by ratioing NADH to FAD content (NADH/FAD). In Previous works, this ratio was shown to discriminate between healthy and tumoral tissues in the bladder^15,16^, breasts^17^, and brain^18^. Other metabolic ratios investigated are the NADH to Porphyrin levels and the Lipopigment to Porphyrin relating metabolic activity with vasculature^18,19^.

The fluorescence lifetime of these fluorophores have also beed studied to discriminate tumor from healthy tissues. The fluorescence lifetime of the NADH measured intraoperatively was found higher in GBM than normal cortex ones^20^, while it was found lower in carcinoma than that found in normal ones^20^. Most studies found in literature focus either on analyzing spectrally-resolved autofluorescence^13,21^ or the temporally-resolved fluorescence^15,16,22^, but few studies tried to implement the complementarity between both methods to better discriminate the different types of brain tissues.

To this end, and to address all these problems cited, we aim to build a multimodal endomicroscope based on non-linear excitation in Near InfraRed (NIR) range where the tissue therapeutic window is located, resulting in better penetration depth and less photodamage. This endomicroscope will be able to perform different fluorescence imaging modalities by detecting several contrasts: 1) Two-Photon Fluorescence Emission (2PEF) imaging of five endogenous fluorophores: NADH, FAD, Lipopigments, Porphyrins I and Porphyrins II; 2) The Fluorescence Lifetime Imaging (FLIM) of these five fluorophores; 3) The autofluorescence spectra and 4) The Second Harmonic Generation (SHG) detection. With two-photon imaging, TPEF/SHG images at the submicron resolution is expected to identify isolated tumor cells that have infiltrated the parenchyma within the field of view, either during open surgery after macroscopically complete surgical removal of the tumor, or during biopsy procedure, using an optic fiber mounted biopsy forceps. Therefore, we hypothesize that tumor microenvironment supporting tumor invasion and infiltration can be differentiated from that of non-infiltrated brain parenchyma.

Although multiscale (DUV to NIR) one and two photon images were acquired from fresh tissue on benchtop systems, our multimodal endomicroscope prototype is currently being developed with imaging capabilities using MEMS mirror scanning technology. This endomicroscope will be able to characterize the nature of all types of human brain tumors through its multimodality in order to give the surgeon fast, accurate and specific information at a subcellular scale on the histological nature of the examinated tissue during the operation. In parallel, and to validate the instrumental development, we are building a big optical database of the fluorescence response of brain tissues in order to explore the different optical signatures of all types of human brain tissues, healthy or tumor, and to characterize each type of tumor with its specific multimodal signature. This database will be used to develop discriminating algorithms giving the surgeon more criteria to make an informed decision.

Most studies focuses on discriminating healthy from tumor tissue^13,21^, however, there are no studies which try to discriminate tumor types from each other, or to define a specific signature for each tumor type and for each grade. In some previous work, we were able to discriminate Grade I from Grade II meningioma through their 2PEF images and their spectral emission analysis^23^. We also managed to discriminate primary from secondary brain tumors using deep UV and NIR excitation^24^. The use of deep UV excitation is particularly efficient in studying the autofluorescence of biomacromolecules due to its high absorption rate in this wavelength range^25^, for example tryptophan, whose fluorescence signal gives an additional marker for monitoring cellular status^25^. Although, our endomicroscope will use visible and NIR excitation, the excitation in deep UV highlights the fluorescence of different fluorophores which can not fluoresce if excited in visible or NIR^25^. Collagen crosslinks emit a fluorescence signal when they are excited in deep UV, while they are a strong source of SHG signal using NIR excitation, indicating the presence of vascularized structures. So, by using different excitation wavelength and adding deep UV excitation, our ability to relate the fluorescence response of the different molecules and to study the alteration of the fluorescence signal of each molecule during cancerogenesis will be improved. All this information will allows us to better follow the metabolic state of the tissue and to find a better discriminative indicator of cancerous tissue.

Many studies were also conducted to discriminate GBM from healthy tissues using different imaging modalities including the autofluorescence over the visible and Near InfraRed (NIR) spectral range^16^, but it is difficult to find a multimodal study which can delineate diffuse LG gliomas from parenchyma or HG from LG gliomas, or to find a specific optical signature for each type by relying on the autofluorescence of brain cells.

For that reason, we have decided to launch a multiscale study in order to discriminate LG from HG glioma and to try to define a specific signature for each grade. This study was performed in collaboration with Sainte-Anne hospital center (Paris, France), providing all fresh samples and with Synchrotron SOLEIL (Saint-Aubin, France), where we have acces to the DISCO beamline platform for deep UV excitation. We performed the NIR measurements using our two-photon multimodal benchtop microscope. We, therefore, present a multimodal study which combines the results and the data obtained from these three imaging platforms. Also included in our study, different molecular ratios analysis and a 3D discrimination algorithm was established for deep UV molecular ratios. Finally, another study was established combining the three molecular ratios derived form different excitation wavelengths in order to discriminate, with high sensitivity and high specifity, the HG, LG glioma from healthy tissues.

## Results

### Results and discussion

#### Deep UV

**Figure 1:**
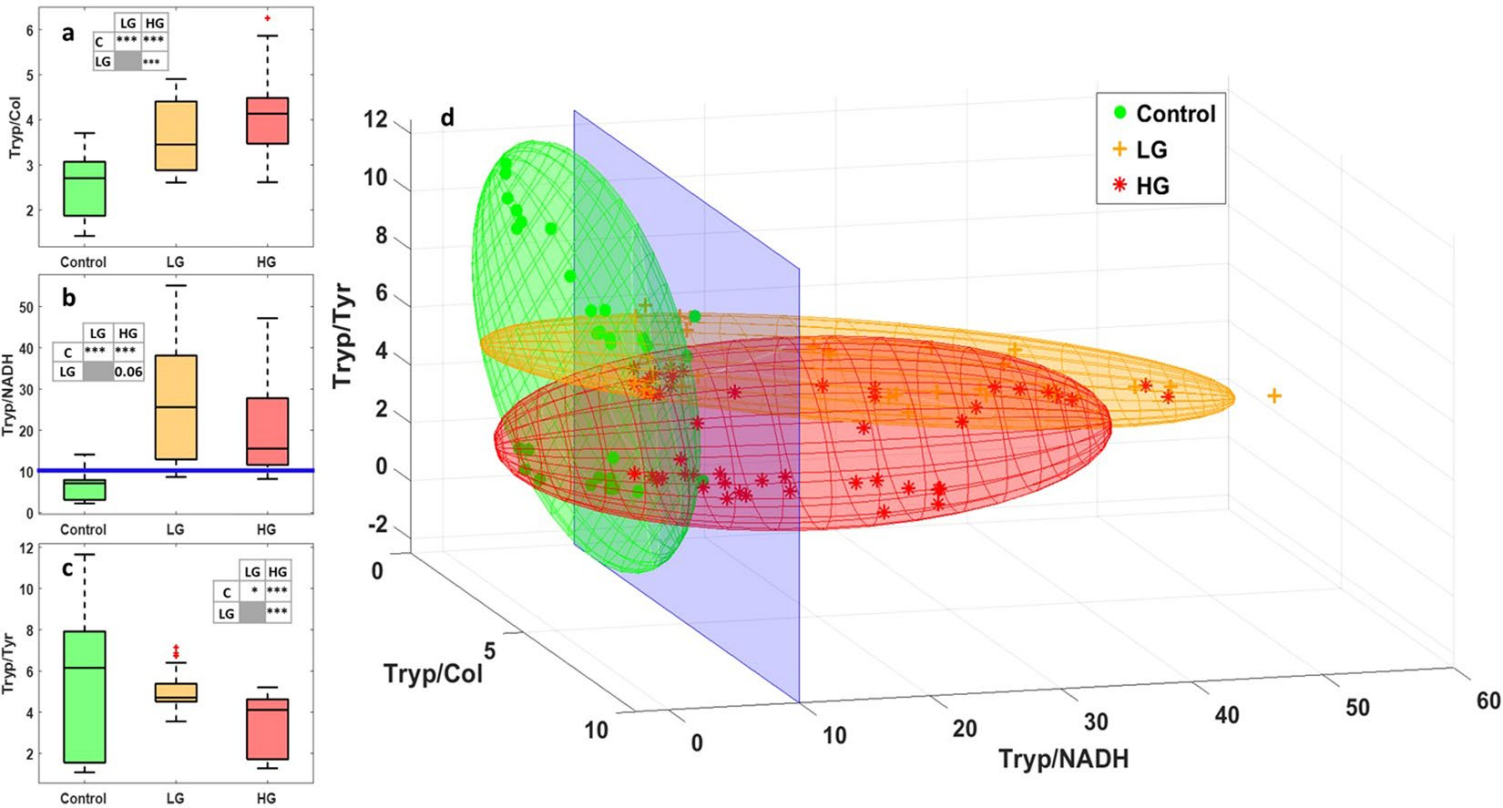
Analysis of the Deep-UV data. Boxplots of three molecular ratios acquired from spectral data under 275 nm excitation wavelength: (**a**) Shows the Tryptophan/Collagen ratio, (**b**) shows the Tryptophan/NADH ratio and (**c**) shows the Tryptophan/Tyrosine ratio for control, LG and HG glioma. The central mark in each box represents the median while the box edges represent the first and the third quartile respectively. The tables recap the p-values corresponding to the t-test of a couple of values distribution (see statistical analysis). (**d**) Represent the 3D-discrimination clouds of the three molecular ratios comparing control, LG and HG glioma.

Concerning the UV spectral measurements, we used spectral measurements for 10 µm thick control and we used 275 nm as an excitation wavelength for LG and HG glioma samples. With these wavelengths, we can excite four molecules: Tryptophan (Tryp), tyrosine (Tyr), collagen (col) and NADH. A fitting process was then done for all obtained spectra to get different molecular ratios data. Figure 1 gathers the main results of the deep-UV study, it shows the possible discrimination extracted based on this excitation range. Three boxplots of three molecular ratios for the tissue types are displayed. Tryptophan/Collagen ratio is shown in Fig. 1(a), Tryptophan/NADH ratio in Fig. 1(b) and Tryptophan/Tyrosine ratio in Fig. 1(c).

As shown, Control tissues were discriminated from tumoral tissues thanks to Tryptophan/Collagen and Tryptophan/NADH ratio, because the control tissues have a lower ratio than LG and HG glioma. Tryptophan/Tyrosine ratio could not discriminate control from tumor, but LG and HG can be separated with this ratio. Based on the observations of these ratios, a discrimination algorithm which combines these three ratios was applied to the spectral data and is displayed in Fig. 1(d). So each point in this 3D graph has three coordinates, which are the three molecular ratios cited above. Each cloud of points was then approximated by an ellipsoid giving an overlap between these groups, as shown in Table 1. As we can see in this 3D plot, control and LG glioma ellipsoid overlap only 4% of the common volume, however, this percentage increases slightly to reach 14% between control and HG glioma ellipsoid. But this percentage increases sharply to reach 46% between HG and LG glioma. So it is clear that this 3D algorithm discriminates between control and tumor tissues very efficiently, while it does not discriminate very well HG from LG glioma.

**Table 1:** Overlap volume precentage between the different ellipsoids.

	LG	HG
Control	4%	14%
LG		46%

#### Visible

**Figure 2:**
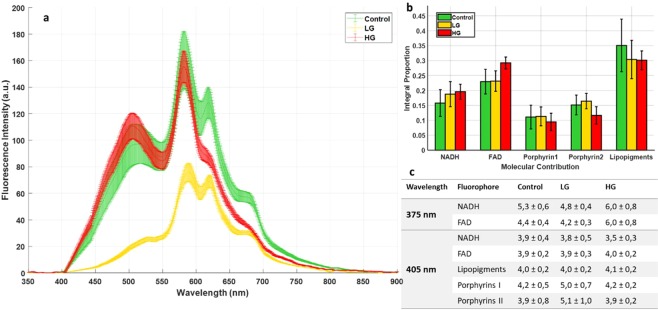
Analysis of Visible data. (**a**) Shows the mean type spectrum of control, LG and HG glioma under 405 nm excitation. (**b**) Shows the Integral proportion of the five endogenous fluorophores: NADH, FAD, Lipopigments, Porphyrins I and Porphyrins II. (**c**) Shows the average lifetime of the five endogenous fluorophores excited in each tissue type at 405 nm and for NADH and FAD excited at 375 nm.

Concerning the visible spectral range, we used spectral and lifetime measurements for our three types of tissues. Figure 2(a) presents the mean emission of fluorescence spectra of control using LG and HG glioma samples under 405 nm excitation wavelength. At this wavelength, we excited five endogenous fluorophores highlighted in our study. Differences were noticed concerning the maximal fluorescence emission intensity of each type, and each type had its specific spectral signature which is interesting. Control presented higher fluorescence emission intensity that overlapped with the HG glioma spectra in the NADH, FAD and lipopigments emission range (centered at 445 nm, 520 nm and 580 nm respectively). This overlapping disappeared in the porphyrins I and II emission range where the HG glioma spectra started to decrease and became lower than control spectrum. The lower fluorescence emission spectrum was observed in the LG glioma. In addition, by calculating the integral proportion of the emission contribution of each fluorophore using our homemade Matlab program^18,26^, we plotted a histogram (Fig. 2b) gathering the contribution of each molecule in the total of each tissue type. Unlike with our previous results on rats^26^ and fixed tissues^18^, when the contribution of all molecules was higher in control tissues, the contribution of NADH and FAD seems to increase with the malignancy of the tissue, while they are lower in control tissues and higher in HG Porphyrins I and II were almost similar to the three examined tissue types with no significant differences, except that the lipopigments are more present in the control samples.

For the fluorescence lifetime measurements, another excitation wavelength of 375 nm was added. At this wavelength, only the lifetime values of NADH and FAD were taken into account. Lifetime values for each molecule at 375 nm and 405 nm are displayed in Fig. 2(c). At 405 nm, the obtained lifetime values of NADH and FAD were similar to the three tissues types. Even the lifetime values of the other molecules were similar, except the lifetime of porphyrins was found to be a bit higher in LG glioma than control and HG glioma. This similarity disappeared in the lifetime values of NADH and FAD at a 375 nm excitation wavelength, where HG glioma presents a higher lifetime value for these two molecules than for control and LG glioma. However, by using 375 nm as an excitation wavelength, we excite more efficiently NADH and FAD than by using a 405 nm wavelength, thus the population of excited NADH and FAD molecules increase, which improves the precision of the results and the obtained lifetime values.

**Figure 3:**
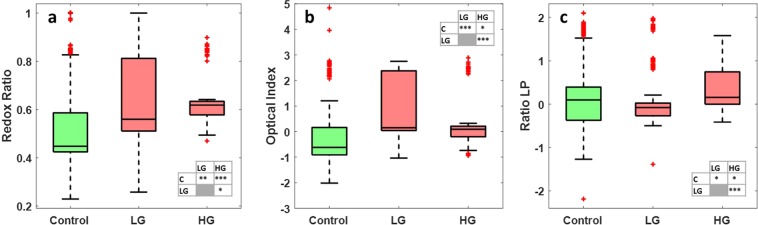
Boxplots of three molecular ratios acquired from spectral data at 405 nm excitation: (**a**) Shows the redox ratio (FAD/(NADH + FAD)), (**b**) shows the optical index (Porphyrins/NADH) and (**c**) shows the ratio LP (Lipopigments/Porphyrins) for control, LG glioma and HG glioma. The central mark in each box represents the median while the box edges represent the first and the third quartile respectively. The values outside the box (red “+”) are considered as outliers. The tables recap the p-values corresponding to the t-test of a couple of values distribution (see statistical analysis).

The last analysis made on the visible spectral data was the exploitation of three molecular ratios. Redox ratio (FAD/(NADH + FAD)), optical index (Porphyrins/NADH) and ratio LP (Lipopigments/Porphyrins) displayed in Fig. 3a–c respectively. Looking at these boxplots, redox ratio and optical index discriminate significantly control tissues from low grade glioma (0.01 < p-value < 0.05 and p-value < 0.01 respectively), while the redox ratio only discriminate control from HG glioma (0.01 < p-value < 0.05). Between LG and HG glioma, only the optical index discriminate these two tissue types (p-value < 0.01).

#### Near Infra-Red

**Figure 4:**
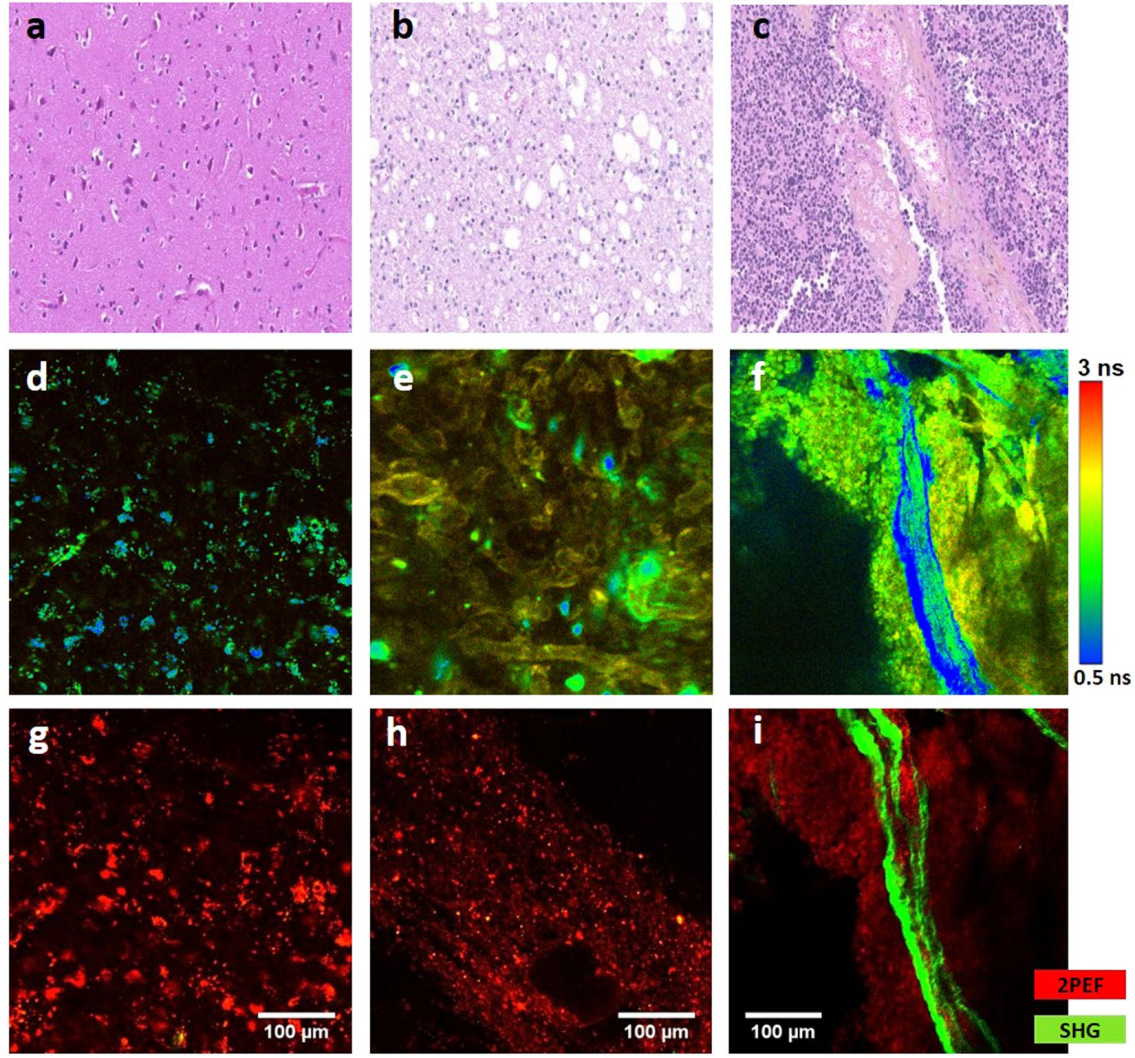
Image comparison through the NIR set-up showing a comparison between control (**a,d,g**), LG (**b,e,h**) and HG (**c,f,i**) glioma using different imaging techniques: Hematoxylin-Eosin staining Images (**a–c**); FLIM images acquired at 890 nm (**d–f**), color bar represents the lifetime value. 2PEF + SHG images at 890 nm showing 2PEF signal in red and SHG signal in green (**g–i**). Scale bar: 100 µm.

The last spectral range in our study was the NIR. The data gathered under NIR excitation reflects the wide multimodality that is achievable through autofluorescence. Figure 4 assembles different images issued from 2PEF + SHG images, FLIM images and a comparison of these two imaging modalities with H&E histological images for each type of samples. This comparison shows the reliability of 2PEF + SHG and mostly FLIM imaging modalities as a diagnosis technique to provide similar information to those acquired from histological H&E images. Similar structures were noticed for each type in these three images. Control samples present a clear H&E image with a low cell density and some small vessels. These similar properties appear in a 2PEF image with strong red spots as well as in FLIM images where we observe spaced green spots. LG glioma presents a high cell density and a compact structure which appears clearly in the FLIM image where we observe a fluorescence carpet with some big spots. Big vessels and strong SHG emission characterize the HG glioma in 2PEF images and the same zone in blue on FLIM images which corresponds to a low fluorescence lifetime. This strong SHG emission can be a good indicator of tumor infiltration in a HG glioma sample, where the dense vascular network support tumor cell invasion.

**Figure 5:**
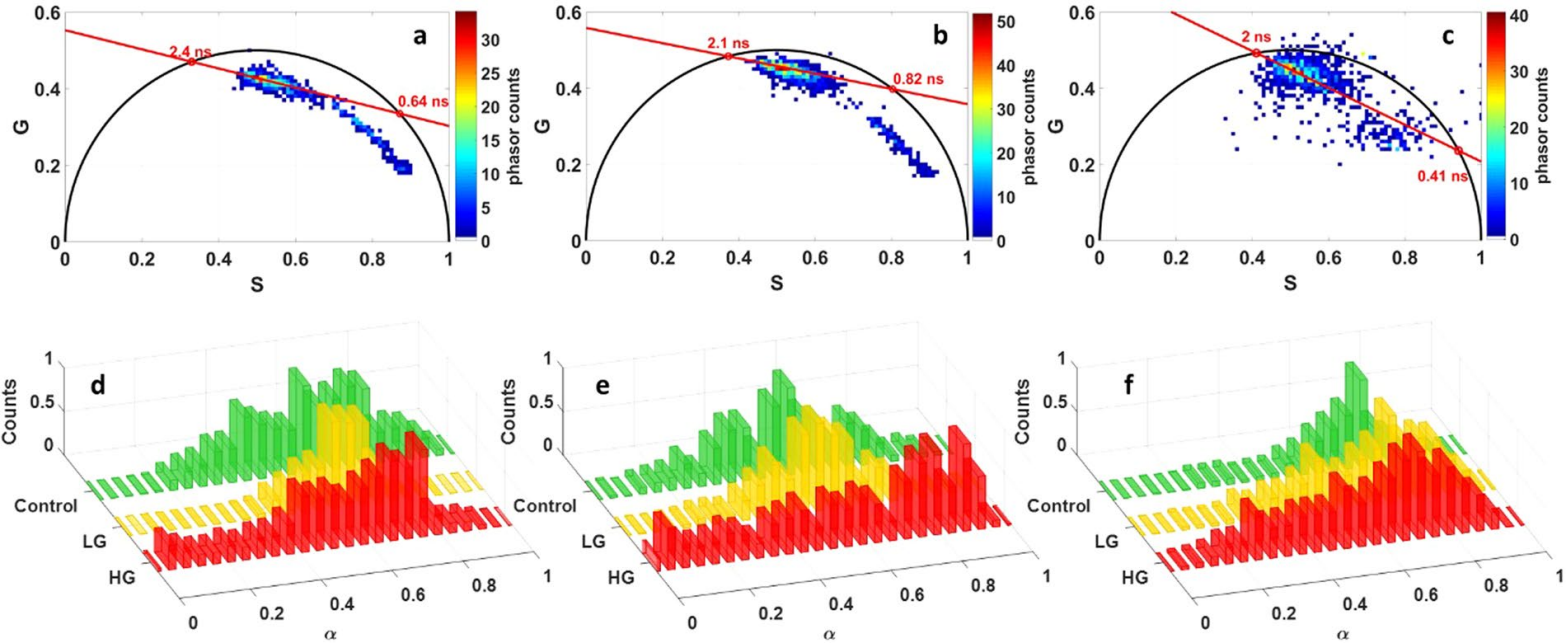
Phasor FLIM Analysis of NIR data. Global histogram of FLIM phasor plot (**a–c**) and Long Lifetime Intensity Fraction distribution (α) for Control, LG and HG (**d–f**) at 810 nm (**b,c,e,f**) at 890 nm (**a,d**) excitation for FAD (**a,b,d,e**) and NADH (**c,f**) channels.

Since FLIM images were clearly differents for each tissue type, we decided to analyse the fluorescence lifetime through a non-fitting technique, the phasor FLIM. The Control, LG and HG glioma fluorescence decay curves taken from FLIM images data were grouped on different global phasor counts (Fig. 5a–c).

The selected FLIM data was divided into two parts: NADH data acquired from channel 1 and FAD data acquired from channel 2. The global phasor FLIM histogram of FAD acquired at 890 nm excitation in all tissue types was plotted and is shown in Fig. 5(a) while that which was acquired at 810 nm excitation was plotted and is shown in Fig. 2(b). Using 810 nm as the excitation wavelength the NADH global phasor FLIM histogram in all tissue types was plotted and is shown in Fig. 5(c). Then these global phasor FLIM histograms were used to plot the histogram of Long Lifetime Intensity Fraction (LLIF) (α) distribution of each molecule in each tissue type. The LLIF of FAD in each tissue type, which is acquired from 890 nm and 810 measurements, is shown in Fig. 5(d,e) respectively. While the LLIF of NADH in each tissue type acquired from 810 nm measurements is shown in Fig. 5(f).

Looking at the FAD LLIF histogram at 890 nm (Fig. 5d), we noticed that HG glioma histogram shifted the most towards the lower LLIF values of FAD at 890 nm, while control samples shifted more toward higher LLIF values. In NADH LLIF histograms at 810 nm (Fig. 5f), we noticed that the contribution of lower LLIF values is low in control samples where its histogram shifts sharply toward higher LLIF values. This contribution starts to be higher in LG glioma and reaches its height in the HG glioma histogram.

**Figure 6:**
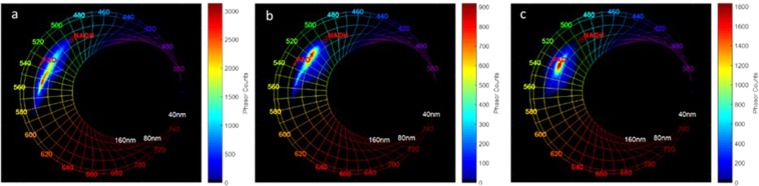
Spectral Phasor Analysis of NIR data at 810 nm excitation wavelength. Comparison of three spectral phasor histogram for: Control (**a**), LG glioma (**b**) and HG glioma (**c**).

Moving to the spectral imaging results at 810 nm, the spectral phasor histogram for each tissue type was plotted and is shown in Fig. 6 for control (Fig. 6a), LG glioma (Fig. 6b) and HG glioma (Fig. 6c). Fingerprints of standard NADH and FAD measured in standard solutions were added to each phasor histogram respectively at 495 nm and 535 nm. The spectral phasor approach is used for spectral unmixing of fluorophore contributions in the spectral images. Regarding the three phasors shown, a maximum wavelength shift between the phasor clouds of each type was noticed. The control spectral phasor counts shifted toward an interval higher than the FAD fingerprint. In the LG glioma it was contained between NADH and the FAD fingerprint, while in HG glioma the phasor cloud was centered and close to the FAD fingerprint.

**Figure 7:**
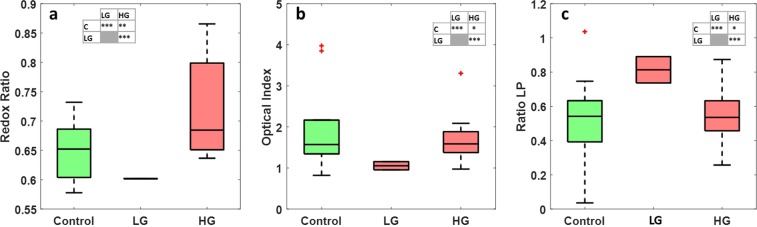
Boxplots of the three molecular ratios acquired from spectral images data acquired at 810 nm: (**a**) Shows the redox ratio (FAD/(NADH + FAD)), (**b**) shows the optical index (Porphyrins/NADH) and (**c**) shows the ratio LP (Lipopigments/Porphyrins) for control, LG glioma and HG glioma. The central mark in each box represents the median while the box edges represent the first and the third quartile respectively. The values outside the box (red “+”) are considered as outliers. The tables recap the p-values corresponding to the t-test of a couple of values distribution. (see statistical analysis).

Finally, molecular ratios were calculated for all tissue types at 810 nm excitation wavelength: Redox ratio (Fig. 7a), optical index (Fig. 7b) and Ratio LP (Fig. 7). Analysing these boxplots, redox ratio discriminates significantly control from HG glioma. Optical index discriminate control from LG glioma, while the ratio LP discriminate control from LG and discriminate LG from HG glioma.

### *UV-VIS-NIR* discrimination

The last analysis consisted of combining three molecular ratios, each issued from a different spectral range in order to plot a discriminative 3D scatter cloud able to discriminate healthy from tumoral tissues, and LG from HG glioma.

**Figure 8:**
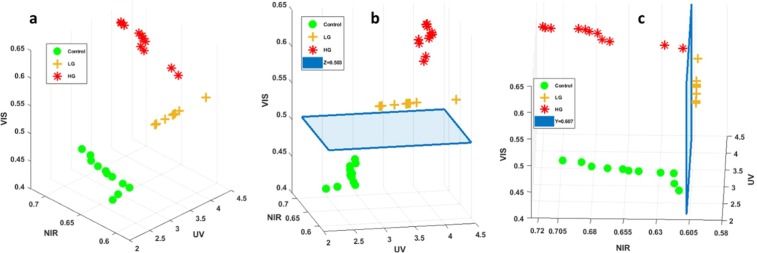
3D scatter cloud of three molecular ratios: Tryp/collagen from UV as X axis (UV), redox ratio from NIR as Y axis (NIR) and redox ratio from Visible as Z axis (VIS). (**a**) General view of the 3D scatter cloud of control, LG and HG glioma. (**b**) Right side view of the 3D scatter cloud showing the Control-tumor threshold plane (z = 0.503). (**c**) Left side view of the 3D scatter cloud showing the HG-LG threshold plane (y = 0.607).

For that we choose Tryptophan/collagen from deep UV spectral range as X axis. Redox ratio from NIR spectral range which as Y axis and redox ratio from visible (VIS) spectral range as Z axis. In order to get a 100% sensitivity, we did not take into account all the molecular ratio values in the distribution of each type, however, we took the median value of each distribution along with the first 5 values that were bigger than and the first 5 values that were smaller than the median value, and using these values we plotted our 3D scatter cloud as shown in Fig. 8a. So in terms of median region values, control LG and HG glioma were clearly discriminated. Figure 8b represents the 3D scatter cloud with a different view angle than Fig. 8a, where a horizontal threshold plane was plotted (z = 0.503). This plane discriminates healthy from tumoral tissues. We acknowledge that if our scatter point is above this plane the tissue is tumoral, and if it is under it, the tissue in healthy. Figure 8c represents also the 3D scatter cloud with another view angle, where a vertical threshold plane was plotted (y = 0.607). This plane was plotted to discriminate LG from HG glioma tissues. We acknowledge that if the scatter point has a NIR redox ratio of less than 0.607, the sample is a LG glioma, and if it has a NIR redox ratio which is bigger than 0.607, it is considered as HG glioma tissue.

## Discussion

In this study, our main goal is to discover, analyse and achieve the best method to discriminate between healthy brain tissues, LG and HG glioma tissues. Relying on the endogenous fluorescence of several molecules in brain cells, different excitation wavelength were used, ranging from deep UV to NIR passing by visible wavelengths. Different imaging modalities on several setups and multiple methods of analysis were performed on our cohort of freshly extracted and fixed brain samples: control samples, low grade glioma and high grade glioma.

In the deep UV spectral range, only few studies have used deep UV excitation to study the endogenous fluorescence emission of human brain tissues. These studies generally compared one or two molecules to find a discrimination between tumoral and healthy tissues^27,28^. Tryptophan being considered as a good internal standard of tissue autofluorescence, the Tryptophan/Collagen and Tryptophan/NADH ratios could be considered as good indicator of cancer proliferation. Indeed, NADH redoxstate and propensity to bound to proteins affects its fluorescence level and was monitored for epithelial cells^29^. Moreover, under near-UV excitation, collagen fluorescence decreases in Barrett’s esophagus versus normal ephitelium^30^. Therefore, even if results obtained from single cells or endocscopy may not be directly related to biopsies results, those two balances (Tryp/Coll and Tryp/NADH) are good indicators of glioma. Pu *et al*.^31^ for example, analysed the Tryptophan and NADH principal components using 300 nm excitation. However, they only showed the preliminary study and used *in-vitro* cells. While Pradhan *et al*. analysed the Tryp/NADH intensity ratio using 310 and 350 nm excitation and found this ratio higher in metastatic cell lines than in non-metastatic and normal cell lines, similar to our findings on glioma tissues. So, we proposed to calculate and analyse three molecular ratios related to the tryptophan molecule: Tryp/NADH, Tryp/tyrosine and Tryp/collagen. Tryp/collagen discriminates control from tumor, when control had a significantly lower ratio (p-value < 0.01), however, Tryp/NADH presents the best reults. A Tryp/NADH threshold value of around 10 could be defined to separate control from tumor (Fig. 1c). Through these results, we built a 3D discriminative algorithm using the ellipsoids and the discrimination threshold value of Tryptophan/NADH ratio (Tryp/NADH), where the three molecular ratios values correspond to the 3 coordinates of each point. We assumed that if the point is inside a tumor ellipsoid (HG or LG) and its Tryp/NADH is above 10, tumors can be detected and confirmed. With a low percentage of volume overlaping between control and tumor elliposids (4% control-LG and 14% control-HG), this test is not able to attribute a value for the percentage of points under 9 and has a sensitivity (Se) of 94% and a specificity (Sp) of 84%.

Concerning the visible spectral range, we conducted spectral and fluorescence lifetime analysis on our cohort. On the one hand, we explored the spectral properties of the fluorescence emission by comparing the molecular contribution of each fluorophore in each tissue type. On the other hand, we preformed a fluorescence lifetime study using six band pass filters to select the specific emission spectral band of each fluorophore to measure the lifetime of each fluorophore separately. Each tissue type seems to have a specific spectral signature. We can particularly underline a difference in the fluorescence intensity between LG and control tissues. The lower fluorescence intensity in LG could be attributed to the angiogenesis, which is a high source of absorption^32^ so less fluorescence is emitted and detected by our setup. The gap between control and tumoral tissues can be noticed as well after 600 nm.

Regarding the proportion of each molecule in the total spectra, the higher value of FAD and NADH in tumorous tissues indicates a higher malignancy. Even though, different studies have shown that the redox ratio decreases in tumor cells as compared to the norm^33,34^, we raised the problem that this ratio seems to decrease between control and low grade but at the same time seems to remain stable between control and glioblastoma. The prevalence of glycolytic pathway over oxidative phosphorylation might not be respected in non-invasive tissues due to the Warburg effect^35^.

Lifetime values are not consistent with the literature on human brain samples^15,16^, however, the values remained in the same order of magnitude. As an example, Butte *et al*.^15,16^ investigated the evolution of the average lifetime in brain tissues as a function of the excitation wavelength. Under 375 nm and 405 nm excitation, they found an average lifetime of around 2.2 ns and 2 ns respectively. It is twice as short as the values that we obtained. Nevertheless, they pointed out the fact that the average lifetime of the three types demonstrated a peak centred at 370–380 nm. We noticed that the average lifetimes are higher in the case of 375 nm excitation than at 405 nm excitation, as well. The Control and Glioma groups did not shown significant differences.

The last and the wider spectral range in term of imaging modalities and analysis is the NIR. We performed three types of measurements on our samples cohort: (1) 2PEF + SHG detection imaging at 890 nm; (2) FLIM imaging at 810 nm and 890 nm and (3) Spectral Imaging at 810 nm excitation wavelength. In addition, H&E histological images were recovered from the neuropathology department in Sainte hospital to compare our images (Fig. 4) with the gold standard technique of histological diagnosis. The similar structures observed in H&E, FLIM and 2PEF + SHG images is very encouraging. In control samples images, the classical cortical arrangement of neurons with a low cell density is visible through clear fluorescent and spaced red spots on the 2PEF and FLIM images. In LG glioma samples, we observed some holes in H&E images due to cysts, a cell density higher than control noticed in 2PEF images and a more compact tissue structure observed in FLIM image. In the HG glioma samples, a very high density of tumor cells, identified in H&E image, surrounds an enlarged vessel observed in 2PEF image by its strong SHG emission (Green) and its lower lifetime in FLIM image (Blue).

The phasor approach shown in this work has the potential to simplify the analysis of FLIM, avoid the fitting errors associated with mono-exponential or multi-exponential analysis, and to give a graphical global view of the processes which affects the fluorescence decays occurring at each pixel in the FLIM image^36,37^.

In the FAD channel, the phasor FLIM histogram at 810 nm and 890 nm excitation wavelengths were similar. Free FAD lifetimes were slightly close (2.4 ns for 890 and 2.1 ns fro 810 nm) but protein-bound FAD lifetimes were different (0.64 ns at 890 nm and 0.82 ns at 810 nm). We observed that the HG glioma LLIF histogram of FAD was the only histogram which is shifted to lower LLIF values. Based on this observation, we assumed that there is more free FAD in glioblastoma tissues than control ones. While in the NADH channel, the calculated free and protein-bound NADH lifetime were respectively 0.4 ns and 2 ns. We also noted that the LLIF repartition shifted to the Lower LLIF values according to the tumor grade. So it seems that the contribution of free NADH increases with the malignancy of the tissue. Thus, it can be assumed that the higher grade gliomas has increases free NADH concentration. All of these results are in line with the study of Skala *et al*.^38^. In their study, FAD and NADH lifetimes were in the same order of magnitude. They also noticed that the proportion of protein-bound FAD and NADH decrease with the grade.

In addition to the FLIM phasor analysis, spectral phasor analysis is applied also to the data issued from spectral images at 810 nm for each tissue type. This technique is a fast and representative way to extract two characteristic parameters from a spectrum that are related to the maximum emission wavelength and the width of the spectrum. It can also be used to separate the contributions of the different fluorophores presented in one fluorescent sample. With this in mind, we decided to apply the method as a standard. NADH (1 mM in Tris Buffer pH = 8.5) and FAD (1 mM in PBS pH = 7.4) were tested and converted as points, respectively at 495 nm and 535 nm on the phasor plot (Fig. 6).

We noticed that these values are red-shifted in comparison with the values in brain tissues we have recovered from the literature. (see spectral fitting). The difference is less significant for FAD than for NADH. despite this, it could be clarified by the spectral red-shift of free form from bound form of the fluorophore^39^. First of all, higher phasor counts on all types (of tissues) which are located on a straight line crossing NADH and 580–600 nm (Lipopigments, Porphyrins). Spectrum shapes are quite different for each tissue type because the peak of low and high grade glioma is closer to NADH, while it is closer and even farther from FAD fingerprint. The lack of information from any previous work using this method, makes a comparison to literature more difficult. Nevertheless, these results are in line with the observations under visible excitation. Furthermore, a widening of the Gaussian ellipsoid and, consequently, of the spectral bandwidth sets the tumoral tissues apart.

Finally, we gathered our molecular ratios results derived from the different excitation wavelength, in order to plot a multiscale discrimination algorithm with a 100% sensitivity able to, one the one hand discriminate control from tumors, and on the other hand to discriminate LG from HG glioma. We, therefore, choose the best disciminator molecular ratio from each spectral range excitation, taking into account the median range values. Tryp/col was choosen from deep UV, redox ratio from Visible and redox ratio from NIR measurements. Then a 3D scatter cloud was plotted (Fig. 8). What we obtained, relying on the median values range of each molecular ratio, is very interesting. Each scatter cloud of each type was separated from every other scatter cloud, which means a 100% sensitivity. A threshold was then defined to discriminate healthy from tumor (z = 0.503) and another one to discriminate LG from HG glioma (y = 0.607). So finally we can admit that if a sample has a visible (VIS) redox ratio <0.503, it is considered as a healthy tissue. If the sample has a VIS redox ratio >0.503 and a NIR redox ratio <0.607 it is considered as a LG glioma, and if the sample has a VIS redox ratio >0.503 and a NIR redox ratio >0.607 it is considered as HG glioma.

Throughout our study, we have brought new approaches to analyze the endogenous fluorescence of different human brain tissue types ranging from deep UV to NIR. Regarding our results, we can conclude that using a large panel of contrast sources is essential in order to achieve the best discrimination between tumor and healthy tissues and between the different grades of the same tumor type. Moreover, this study highlights numerous discrimination criteria between healthy and different grade tumor tissues. The combination of different quantitative and qualitative measurements of fluorescence microscopy allowed us to reach this goal. But there is still a lack in the number of collected data. A larger database would be crucial in the future in order to build a more robust analysis program with different discrimination thresholds and particularly through NIR-Visible molecular ratios where the set of values of these ratios was not robust enough to discriminate control from tumor and to offer a sensitivity and a specificity higher than 80%.

We are currently enriching our optical database with more biopsied samples. A sample size larger than 50 was enough to differentiate between primary and secondary brain tumors, with a sensitivity of 97% and a specificity of 100% based on the Optical Index ratio, the redox ratio and the mean lifetime value^26^. Since the morphological and metabolic difference in low grade glioma vs. normal and low grade glioma vs. high grade glioma is more subtle (Fig. 4, for instance), we expect that a sample size larger than 50 is necessary to obtain clinically useful statistical significance. In fact, a power analysis was performed for the three ratios used in the UV-visible-NIR study to determine the sample size necessary to test whether these ratios (Tryptophan/Collagen, visible redox ratio, and NIR redox ratio) were statistically different for each pathology by setting the type I and II error at 0.05 and 0.2 respectively^40^. Interestingly, the Tryptophan/Collagen ratio required the lowest number of samples to reach statistical significance, while the visible redox ratio needed the highest number of samples. Specifically, 107 samples were required to compare healthy tissue with LG glioma, while only 35 samples were needed to compare healthy tissue with HG glioma. As one would expect, more samples are needed to discriminate between HG and LG glioma, specifically 118 samples. For this reason, the sensitivity and specificity values were not calculated for the NIR and visible data.

The final objective is to apply this technique in real-time during surgical resection, so that the tissue can be analyzed immediately in a way to facilitate the resection. This study brings us closer to our goal and opens the door for a pilot *in vivo* study.

In tandem to constructing an optical database based on one- and two-photon fluorescence excitation and on multiscale detection, a nonlinear endoscope prototype is being optimized with imaging capabilities. The custom-built fibered endoscope is already capable of generating excitation pulses below 100 fs delivered to the distal end of a 5 meter long microstructured double-clad photonic crystal fibre^41^. A miniature MEMs system is being incorporated to extend the endoscope’s capabilities to perform spatially- and depth-resolved imaging. Visible and NIR excitation is possible with the non-linear endoscope and thus results pertaining to both regimes can be directly translated to future *in vivo* measurements, where NADH, FAD, lipopigments and porphyrins I & II are the molecules of interest. DUV excitation on the otherhand, was useful to highlight the properties of other endogenous fluorophores that play a role in malignant transformation such as collagen and tryptophan. Although, the enhanced SHG signal observed in brain tumors is known to result from increased disorganization of collagen fibers in the extracellular matrix facilitating invasion, spectral analysis of the autofluorescence spectra under DUV excitation^24^ informed us that collagen levels themselves are elevated according to tissue grade. This further supports the hypothesis that the high SHG signal in Fig. 4i signifies tumor infiltration.

So in summary, the three excitation regimes provided complimentary properties that histologically characterize healthy brain tissue, low and high grade glioma. The quantitative parameters derived from visible and NIR excitation will be directly measured with the multimodal endomicroscope so that *in situ* tissue discrimination beyond the solid tumor will be possible. This is paramount for delineating diffuse LG, which is not feasible under conventional 5-ALA fluorescence. Specifically, since the endomicroscope is equipped with two-photon imaging, TPEF/SHG images at the submicron resolution is expected to identify isolated tumor cells that have infiltrated the parenchyma within the field of view, either during open surgery after macroscopically complete surgical removal of the tumor, or during biopsy procedure, using an optic fiber mounted biopsy forceps. Additionally, tumor microenvironment supporting tumor invasion and infiltration can be differentiated from that of non-infiltrated brain parenchyma by the former’s high SHG signal from dense vasculature.

## Materials and Methods

### Samples

Samples were collected from the Neurosurgery and Neuropathology department at Sainte-Anne Hospital upon the approval of the Sainte-Anne Hospital – University Paris Descartes Review Board. (CPP Ile de France 3, S.C.3227). All methods were carried out in accordance with relevant guidelines and regulations. Informed consents were obtained from all the samples coming from human subjects whose clinical data were recorded (Table 2). Different diffuse glioma grades were compared to control samples extracted during epileptic surgery. These samples were maintained in normal saline solution to avoid desiccation, in a temperature-controlled dark room dedicated to optical imaging. Firstly, these samples were studied on a visible setup at the CHSA Platform then on a nonlinear multimodal setup at the PIMPA Platform. The whole process took less than two hours. After this procedure, the fresh samples were fixed with 4% paraformaldehyde, embedded in paraffin and stained with H&E then digitized using Digital Slide Scanner NanoZoomer 2.0 (Hamamatsu Photonics K.K, Hamamatsu, Japan).

**Table 2:** Clinical Characteristics of our samples cohort.

Sample	Number	Mean of patients age
Control	16	30.6
Low grade glioma	6	35
High grade glioma	14	56.6

Secondly, these samples were stored at −80 °C after being freshly analysed. Few hours before cutting, the tissues were put at −20 °C, after which they were cut at −18 °C into 10 μm slices using a cryostat (CM 1950, Leica Microsystems). The tissues were then fixed with ethanol at 100 °C and stored at 4 °C until experimentation. The 10 μm fixed slices were then used at the PIMPA Platform and on Deep UV setup at the DISCO Platform.

### Deep UV setup

The deep UV spectral data have been acquired on DISCO beamline at Synchrotron Soleil (Saint Aubin, France), where we performed full field imaging and spectral measurements using 275 nm as an excitation wavelength on 10 µm thick samples. Using this excitation wavelength, we were able to excite four different molecules: Tyrosine, Trypthophan, collagen and NADH. The setup details were described in a previous experiment conducted by our team^24^. For each sample, measurements were conducted on 1 to 4 selected zones (depending on the sample volume). Therefore, the data issued from each zone were taken into consideration separately.

### Visible setup

A detailed description of the visible setup has been published elsewhere^42^. Located at Sainte-Anne Hospital (CHSA), this setup consist of a bi-modal endoscope which uses 375 and 405 nm as the excitation wavelengths. Spectral and lifetime measurements were performed on freshly extracted tissues. In this range we excited five fluorophores, NADH, FAD, Lipopigments and Porphyrins I and Porphyrins II. Spectral acquisition was accomplished for several longitudinal lines of each sample, while the lifetime measurements accomplished five different Regions Of Interest (ROI) for each sample. Measurements for each sample were taken between 10 to 15 minutes.

### NIR setup

The NIR multimodal optical setup was described in our previous work^14,23^. It consists of a two-photon multimodal benchtop microscope (Leica SP8, Leica microsystems) able to perform four different optical imaging modalities: (1) One and two-photon fluorescence imaging (2) two-photon FLIM measurement, (3) SHG imaging and (4) one and two-photon Spectral imaging.

Analyses were conducted using the dedicated Leica software (LAS-X) as well as Matlab and imageJ. For FLIM measurements, 3 * 3 images mosaic per sample were analyzed using 810 and 890 nm the excitation wavelengths. The same technique was used also for 2PEF and spectral imaging, where 2PEF images were acquired using 890 nm and the spectral images using 810 nm. The collected fluorescence signal is divided into two detection channels. Each detection channel contain a super sensitive hybrid non descanned (HyD NDD) detector. In the first detection channel (channel 1), a bandpass filter with a bandwidth of 448 ± 20 nm (FF01-448/20-25, semrock, USA) was used to select the NADH fluorescence signal, and in the second detection channel (channel 2) another bandpass filter with a bandwidth of 520 ± 35 nm (FF01-520/35-25, Semrock, New York, USA) was used to select the FAD fluorescence signal.

### Data analysis

#### Spectral fitting and molecular ratios analysis

Spectral data processing was conducted using a new homemade matlab interface whose development is based on matlab data processing codes used in previous work by our team^18,26^.

**Figure 9:**
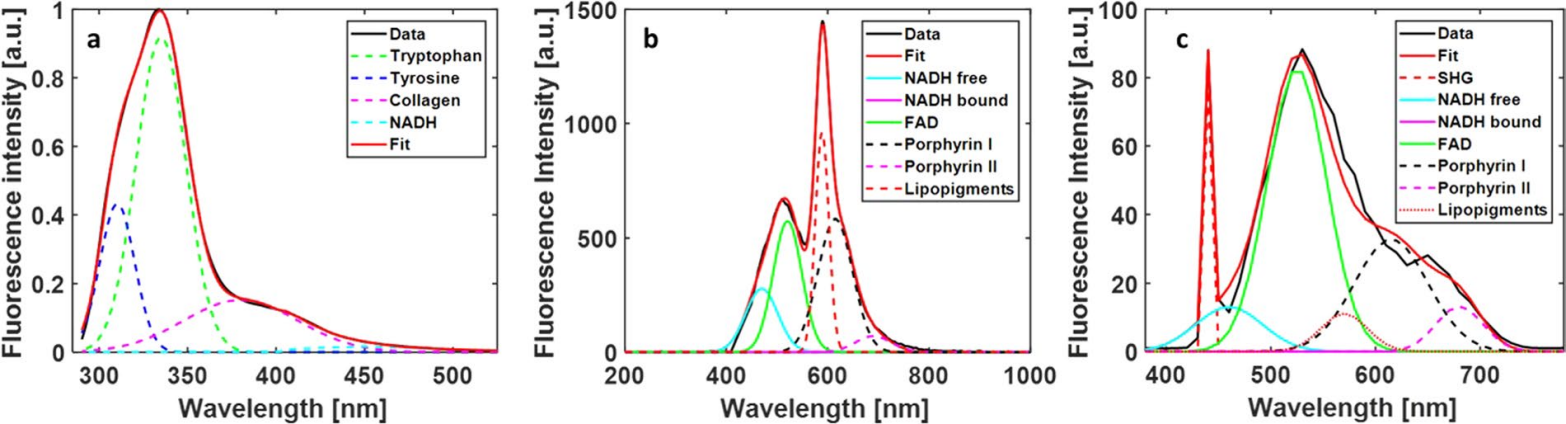
Fitting examples of the spectral data collected during Deep-UV excitation (275 nm) (**a**), for Visible excitation (405 nm) (v) and for NIR excitation (890 nm) (**c**).

Excitation at 275 nm highlight the fluorescence emission of four fluorophores: tyrosine (tyr), tryptophan (tryp), collagen (col) and NADH. Each emission was represented by a Gaussian fit (Fig. 9a) whose maximum wavelength and bandwidth follows the given values as shown in Table 3.

**Table 3:** Gaussian Parameters taken into account to fit the fluorescence emission spectrum of each fluorophore excited with 275 nm.

Fluorophores	Maximum wavelength (nm)	Spectral bandwidth (nm)
Tyrosine	301–311	0–50
Tryptophan	335–345	0–10
Collagen	380–420	0–50
NADH	430	0–60

On the other hand, the visible excitation at 405 nm and the two-photon excitation at 810, highlight the fluorescence emission of five fluorophores: NADH (Free and protein bound), FAD, lipopigments, porphyrin I and porphyrins II. These molecules were chosen through a literature review^11^. Six components were fitted by Gaussian curves thanks to this review (Fig. 9b,c). A recapitulation table of the Gaussian parameters is given in Table 4.

**Table 4:** Gaussian Parameters used to fit the fluorescence emission spectrum of each fluorophore excited with 405 nm.

Fluorophores	Maximum wavelength (nm)	Spectral bandwidth (nm)
NADH free	460–470	45–50
NADH bound	443–445	40–48
FAD	520–530	30–50
Lipopigments	570–600	0–180
Porphyrins I	615–630	0–10
Porphyrins II	675–690	0–10

In addition, and for all spectral data, the integral proportion of each endogenous fluorophore was then determined: it is defined by the ratio of the integral under the emission curve of the fluorophore contribution on the integral under the curve of the total fit. spectrum. Using this type of analysis, we were able to calculate different molecular ratios.

For deep UV measurements, three molecular ratios were extracted:

$${\rm{Tryp}}/{\rm{Tyr}}=\frac{{\rm{Tryptophan}}}{{\rm{Tyrosine}}};\,{\rm{Tryp}}/{\rm{NADH}}=\frac{{\rm{Tryptophan}}}{{\rm{NADH}}}\,{\rm{and}}\,{\rm{Tryp}}/{\rm{coll}}=\frac{{\rm{Tryptophan}}}{{\rm{Collagen}}}$$

For visible (405 nm) and for NIR (810 nm) spectral measurements, other three molecular ratios were extracted:

$${\rm{Redox}}\,{\rm{ratio}}=\frac{{\rm{FAD}}}{{\rm{NADH}}+{\rm{FAD}}};{\rm{Optical}}\,{\rm{index}}=\frac{{\rm{Porphyrins}}}{{\rm{NADH}}}\,{\rm{and}}\,{\rm{Ratio}}\,{\rm{LP}}=\frac{{\rm{Lipopigments}}}{{\rm{Poprphyrins}}}$$

#### Spectral phasor

The NIR spectral images at 810 nm excitation were treated and extracted through ImageJ©. In the spectral phasor analysis, the fluorescence emission signal of each pixel in the image was reduced to a “phasor” that is made up of two numbers: the real and imaginary parts of the Fourier transform of the fluorescence signal. These two numbers are used as coordinates in a histogram plot, known as the phasor plot. The grid of the figure covers the maximum emissions from the spectral range of 380–780 nm by 10 nm steps, and the spectral width varies from 20 to 100 nm by steps of 20 nm each. Using this method, we obtain one histogram for each sample imaged on the NIR setup. A global type histogram was plotted afterwards by adding all the histograms belonging to the same type.

To obtain a better description and a better analysis of our spectra, we measured under the same conditions the fluorescence emissions of two standard fluorophores in solution: NADH (,1 mM in Tris Buffer, pH = 8.5, Grade I, disodium salt, ref: 10107735001 sigma aldrich) and FAD (1 mM in PBS, pH = 7.4, Flavin adenine dinucleotide disodium salt hydrate ≥95% (HPLC), powder, F6625, sigma aldrich). These measurements facilitates the tracking on the phasor, with one point and one 95%-confidence Gaussian ellipsoid for each standard.

#### Fluorescence lifetime and phasor FLIM

Fluorescence lifetime measurements were realized using the Time Correlated Single Photon Counting (TCSPC) technique. For visible lifetime measurements data, lifetime decay curves were adjusted by a mono-exponential fit using fluofit software (PicoQuant, GmbH, Berlin, Germany). For an acceptable fit, the two criteria taken into account were: (1) 0.8 ≤ χ^2^ value ≤ 1.2 and (2) residuals distribution around 0 within the interval +4 and −4.

In NIR, FLIM images data were acquired via Symphotime software (PicoQuant, GmbH, Berlin, Germany), the phasor technique was used to treat this data. The procedure of plotting the figure started by collecting the fluorescence decay curve of each pixel of a FLIM 3 * 3 images mosaic of each sample, so for each sample we had nine FLIM images. For each FLIM image we selected, for 810 nm excitation, the signal of FAD filter (520 ± 35 nm) and the NADH filter (448 ± 20 nm), while for 890 nm we only selected the signal of FAD filter. Each image was also treated and extracted using ImageJ©. Each FLIM image is constituted of 512 * 512 pixels, each pixel containing one decay curve. We reduced the size of each image from 512 * 512 to 16 * 16 pixels. So the new reduced pixel have a real size of 32 * 32 = 1024 pixels. The decay curves of each 1024 pixels were added together to obtain one decay curve I(t).

The phasor approach was used to treat lifetime data where the decay curve I(t) of each reduced pixel is represented in a graphical view. This approach works in a new “space”, each decay is represented by a unique vector, called phasor, in the “phasor plot”, and it have its unique location in the phasor plot^36,37,43^.

Following the Eqs (1) and (2), each decay I(t) is converted into two coordinates in a Cartesian plot:

1 $${{\rm{S}}}_{{\rm{i}}}({\rm{\omega }})=\frac{{\int }_{0}^{\infty }\,{\rm{I}}({\rm{t}})\cos ({\rm{\omega }}{\rm{t}}){\rm{dt}}}{{\int }_{0}^{\infty }\,{\rm{I}}({\rm{t}}){\rm{dt}}}$$

2 $${{\rm{G}}}_{{\rm{i}}}({\rm{\omega }})=\frac{{\int }_{0}^{\infty }{\rm{I}}({\rm{t}})\sin ({\rm{\omega }}{\rm{t}}){\rm{dt}}}{{\int }_{0}^{\infty }{\rm{I}}({\rm{t}}){\rm{dt}}}$$

where, Si(ω) and Gi(ω) correspond respectively to the x and y coordinates of the phasor, and the index “i” identify a pixel of the image; ω is the laser repetition angular frequency which is related to the sampling period (Ts) and to the signal length (L) using the Eq. (3):

3 $${\rm{\omega }}=\frac{2{\rm{\pi }}}{{\rm{L}}\,{{\rm{T}}}_{{\rm{s}}}}$$

For each reduced pixel, we acquire Si(ω), Gi(ω) and Mi: the normalized integration under the decay curve. These three sets of numbers provided the phasor histogram with the initial reduced 3 * 3 mosaïc image. Each reduced pixel of the FLIM image gives a point (or a count) in the phasor plot.

After this procedure, the global phasor histogram was plotted. It grouped the phasor counts of all the samples and for each selected molecule. Then, the local maxima of the histogram was identified in order to draw the best fitting line. The two intersections between this line and the circle segment are linked to the two fluorescence lifetimes values^43^. A projection on the line of each phasor count (vector) was accomplished to calculate, for each type of tissue, the Long Lifetime Intensity Fraction (LLIF) marked with a symbol alpha (*α*)^43^ in Fig. 5, in order to estimate the similarities or the dissimilarities between the different tissue types. This parameter helps us to determine the fraction of fluorescence emitted by each component presented in our excited zone.

#### Deep UV discriminative algorithm

Three molecular ratios (Tryptophan-Collagen, Tryptophan-NADH and Tryptophan-Tyrosine) were presented in the 3D-scatter plot of deep UV measurements for the three groups of tissues (Fig. 2). The scatter cloud of each group was approached by a Gaussian ellipsoid using the mean and the standard deviation as parameters for the covariance with the ellipse to cover 60% of the total probability mass. The overlap volume between the three ellipses was then reported as a percentage to assess the performance of such an algorithm. A blind analysis was conducted, and the sensitivity (Se) and the specificity (Sp) of the discrimination criteria were calculated using the Eqs (4) and (5) cited below:

4 $${{\rm{S}}}_{{\rm{e}}}=\frac{{\rm{TP}}}{{\rm{TP}}+{\rm{FN}}}$$

5 $${{\rm{S}}}_{{\rm{p}}}=\frac{{\rm{TN}}}{{\rm{TN}}+{\rm{FP}}}$$

where:

TP: True Positive, defined as tumoral tissue classified as tumoral.

FP: False Positive, defined as control tissue classified as tumoral.

TN: True Negative defined as control tissue classified as healthy.

FN: False Negative, defined as tumoral tissue classified as healthy.

### Statistical analysis

A student test (t-test) was performed for all molecular ratios data series to analyse statistically the different indicators statistically in order to obtain a p-value with a criteria of significance. On each boxplot figure, a table with stars “ *” icon indicator was displayed showing the p-value obtained comparing each couple of tissue type, where:

*Correspond to a p-value > 0.05.

**Correspond to 0.01 < p-value < 0.05.

***Correspond to a p-value < 0.01.

A p-value < 0.05 was considered as discriminating for two data series of two different tissue types.
